# Causal relationship between osteoporosis and osteoarthritis: A two-sample Mendelian randomized study

**DOI:** 10.3389/fendo.2022.1011246

**Published:** 2022-10-21

**Authors:** Liu Lin, Pan Luo, Mingyi Yang, Jiachen Wang, Weikun Hou, Peng Xu

**Affiliations:** Department of Joint Surgery, HongHui Hospital, Xi’an Jiaotong University, Xi’an, Shaanxi, China

**Keywords:** osteoporosis, osteoarthritis, Mendelian randomization, genetics, causal relationship

## Abstract

**Introduction:**

At present, clinical studies have confirmed that osteoporosis (OP) has an inverse relationship with osteoarthritis (OA), but it has not been proven from the point of view of genetics, so our study hopes to clarify the potential effect of OP on OA at the level of gene prediction through two-sample Mendelian randomization (MR) analysis.

**Methods:**

A two-sample MR was adopted to research the causal relationship of OP with OA (including total OA, knee OA and hip OA). All data come from a public shared database. Such traditional methods as simple and weighted models, inverse variance weighted, weighted median, and Mendelian Randomization (MR-Egger) regression were employed to assess the causal effect of OP on OA. We used the Pleiotrophy RESidual Sum and Outlier (MR-PRESSO) method and MR-Egger method to study sensitivity. The leave-one-out test is used to determine the influence of outliers. The heterogeneity was calculated by using Cochran Q statistics and MR-Egger regression in the inverse variance-weighted (IVW) method. *P* > 0.05 indicates that there is a large heterogeneity. MR-Robust Adjustment Profile Score (RAPS) is stable to both systematic and specific multiplicity, so we used MR-RAPS as a supplementary method to verify the results of IVW.

**Results:**

According to the results of IVW, we found that there was a causal relationship between OP and total OA, and OP reduced the incidence of total OA (beta=-0.285, OR=0.751, *P* value< 0.016). The MR estimation of the causal effect of OP on knee OA suggested that the genetic prediction of OP was negatively correlated with knee osteoarthritis (KOA) (IVW: beta=-6.11, OR=0.002, *P* value< 0.016). The IVW results suggested that OP was causally related to hip OA, and OP had a protective effect on hip OA (beta=-5.48, OR=4.15e-3, *P* value= 3.99e-3). Except for heterogeneity in the analysis of OP and knee OA, there was no horizontal pleiotropy or heterogeneity in the other analyses.

**Conclusion:**

We explored the causal relationship between OP and OA through a two-sample MR analysis and found that OP can reduce the incidence of OA (including knee OA and hip OA).

## Introduction

Osteoarthritis (OA) is the main cause of inconvenience for elderly individuals. With the ageing of the population, an increasing number of adults are expected to be affected by arthritis in large joints, such as knee OA (KOA) and hip OA (HOA) (1). OA mostly causes morning stiffness, joint pain and tenderness, joint swelling or deformity and then reduces the quality of life of patients (2). Risk factors that have been identified to cause OA (3) consist of age, sex, mechanical factors, genetic susceptibility, and obesity.

As a systemic skeletal disease that commonly occurs in people, osteoporosis (OP) is associated with ageing, characterized by declining bone strength, deteriorating bone tissue microstructure, and subsequently increased risk of fracture (3). At present, clinical studies have confirmed that OP has an inverse relationship with OA (4), and bone mineral density (BMD) has been proven to be positively correlated with the risk of KOA and HOA (5, 6). However, although some longitudinal studies have found that higher axial BMD is associated with an increased risk of knee joint OA when OA is defined by Kellgren-Lawrence (K-L) classification, there is no definite relationship when OA is defined as joint space narrowing (JSN) (7). Compared with OP group, the bone mass and bone mass of OA patients were not only different, but also the contents of growth factors (such as IGF and TGF β) needed for bone repair were increased (8). These general bone features of OA bone may explain the reverse relationship of OA-OP. However, the relationship between OP and OA has not been proven from a genetic point of view.

Genome-wide association research (GWAS) has made a significant contribution to the identification of SNP associated with common complex diseases (9). It can understand the genetic basis of many complex traits in common human diseases (10). Mendelian randomization (MR) analysis examines any association between risk factors and disease outcomes by using genetic variation as an instrumental variable (IV) (11). Since genotype precedes the progression of the disease and is largely independent of postnatal lifestyle or environmental factors, this technique can minimize confusion and avoid reverse causality bias (12). For example, Fernando et al. found that CRP has a protective effect on the risk of schizophrenia through Mendelian randomized analysis (13). Based on the existing genetic database, the gene variants that control the pathogenesis of OP can be regarded as IVs to further study the effect of OP on the risk of OA. Therefore, our study hopes to clarify the potential effects of OP on OA at the genetic level by the two-sample MR method.

## Methods

We used two-sample MR to study the causal relationship of OP with the risk of OA and its subtypes. All data come from a public shared database and do not require the consent of the participants.

### Data sources

Data on genetic variations associated with OP were taken from a genome-wide association studies (GWAS) that collected 7547 OP samples and 455386 control samples saved in the UK Biobank (UK Biobank: 20002#1309) to identify genetic mutations. As a large-scale constructive cohort study, the UK Biobank stores samples from approximately half a million people aged 37 to 76 from across the UK (14).

There are three sources of OA data in this study, including total OA, KOA and HOA. The GWAS data of total OA (self-reported OA at any site) were derived from the UK biobank (UK Biobank: 20002#1465), with a total of 38472 OA patients and 424461 control samples. KOA GWAS data were obtained from a previously published study (15). The study included 24955 KOA samples and 378169 control samples. The HOA data were also derived from a published study (15), which included 15704 HOA patients and 378169 controls. All participants were of European ancestry. Detailed data and statistics about the samples, genotyping and imputation are accessible in published studies (15).

### Genetic variants

To obtain stable estimates, MR requires strict adherence to three assumptions: (1) the genetic variants included in the analysis are significantly associated with exposure; (2) as an instrumental variable of exposure, the data should be independent of confounding factors associated with selected exposures and outcomes; and (3) the outcomes are supposed to be influenced by the genetic variation not through other biological pathways but merely through exposure (in other words, no horizontal pleiotropy effect is allowed). Single nucleotide polymorphisms (SNPs) are independent genetic predictors and are regarded as IVs when they satisfy three strict assumptions (16). For every SNP independent variable, namely, those not in linkage disequilibrium, we set a genome-wide significance threshold *P* value (*P*<5×10^-8^), a linkage disequilibrium correlation coefficient r^2^ (r^2^<0.001), and a number of bases between two SNPs (kb >10000), and further quality control was based on a minor allele frequency>1%. Then, we removed variants with F values (calculated as *F*=*β* ^2^_*exposure*_/*SE* ^2^_*exposure*_ )<10 and SNPs with incompatible alleles (17).

### Statistical analyses

We used the above data to determine the correlation between OP and OA and its subtypes by the two-sample MR method. The aetiological effects of OP on OA were estimated by the method of traditional inverse variance weighted (18), Mendelian Randomization (MR)-Egger regression, the weighted median approach, and simple and weighted models (19, 20). If all included SNPs satisfy the assumption of being a valid tool variable, IVW can provide accurate estimates (21). So when there are no weak IVs, we take IVW as the main result. We use the MR- Pleiotrophy RESidual Sum and Outlier (PRESSO) method and MR-Egger method to study sensitivity. MR-RAPS can provide a stable result for heterogeneity and multiplicity analysis and can provide a stable inferring basis for MR analysis through many weak instruments. Therefore, we recommend that Robust Adjustment Profile Score (RAPS) estimators often be used in practice, particularly on occasions where complex features exist with both exposure and outcomes (22). Therefore, we use MR-RAPS as a supplementary method to verify the results of IVW. We use the leave-one-out test to determine the influence of outliers. The heterogeneity was calculated by using Cochran Q statistics and MR-Egger regression in the IVW method, and the *P* value was 0.05, indicating that there was a large heterogeneity. The threshold of the *P* value corrected by Bonferroni (*P*< 0.016) was used to indicate the statistical significance of the preliminary analysis. The threshold of *P*< 0.05 was used in all sensitivity analyses. We used the Mendelian Randomization and TwoSampleMR packages in R version 4.1.2 for analysis.

## Results

After the SNP of the incompatibility allele was removed, the details of all independent SNPs associated with exposure are shown in **Supplementary File 1**. A total of 16 independent SNPs associated with OP were identified. In our study, the F statistics of IV related to exposure are all greater than 10, indicating that the possibility of variable deviation of weak tool variables is very small.

### The causality between OP and total OA

**Figure 1:**
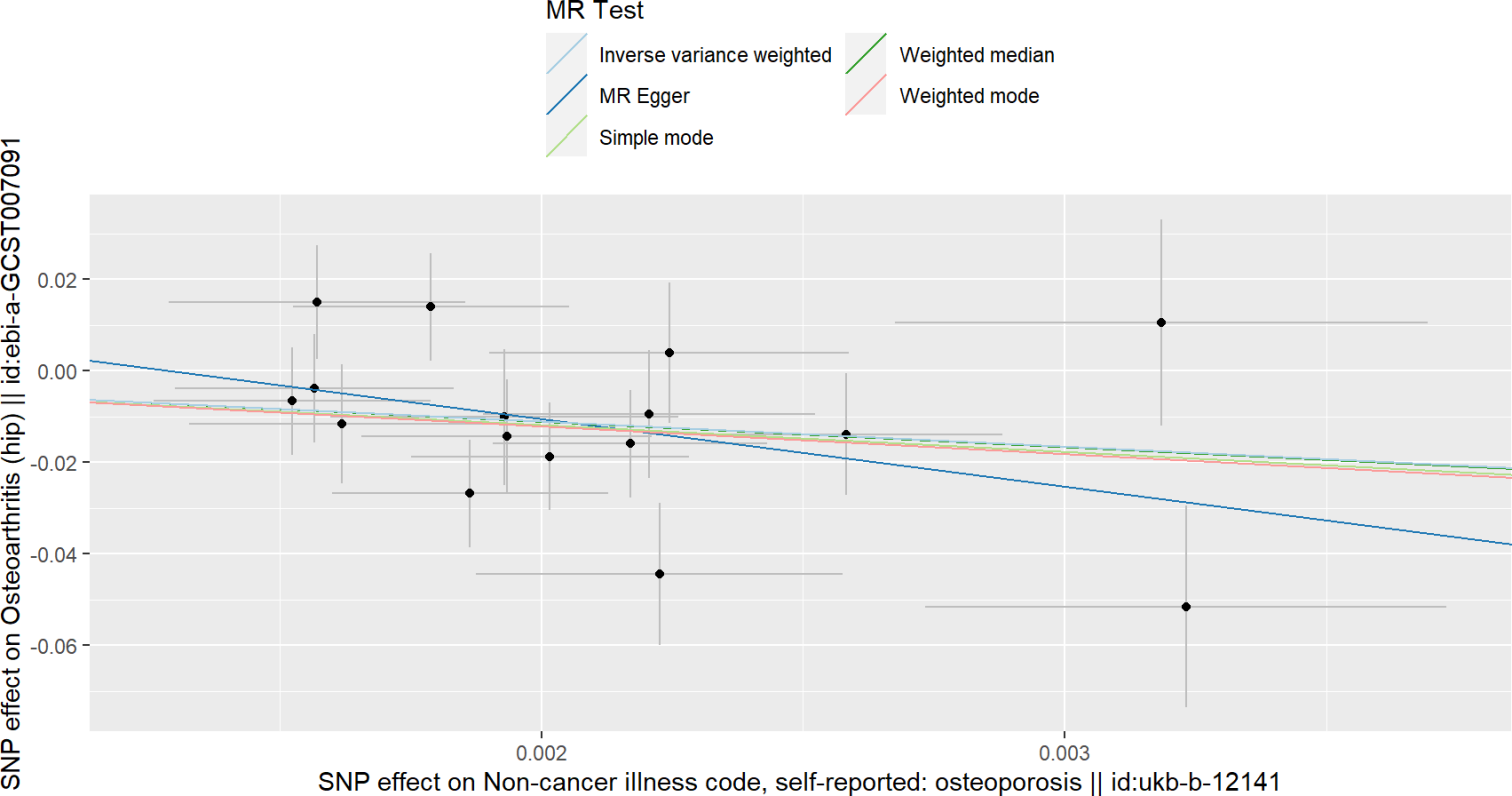
Scatterplots of the causal relationships between osteoporosis and total osteoarthritis.

As shown in **Table 1**, according to the results of IVW, we found a causal relationship between OP and total OA, and OP reduced the incidence of total OA (beta=-0.285, OR=0.751, *P* value< 0.016) (**Figure 1**). In addition, the results of weighted median and MR-RAPS are consistent with those of IVW (**Table 1**). However, the results of Weighted mode, Simple mode and MR-Egger were not significant (**Table 1**). The results of MR-PRESSO analysis, MR-Egger intercept analysis and Cochran’s Q test are displayed in **Tables 2**, **3**. The P values of the three tests were all greater than 0.05, which means that there was no heterogeneity or pluripotency in this study. The scatter plot is shown in **Figure 1**. The forest map of this analysis is shown in **Supplementary File 2**; **Figure S1**. We did not find problematic SNPs through the leave-one-out test, as shown in **Supplementary File 2**; **Figure S4**. According to the above results, since no horizontal pleiotropy exists in the analysis, the results of IVW should be regarded as the main criterion of causality, so we think that OP is a protective factor against total OA.

**Table 1:** MR estimates from different methods of assessing the causal effect of osteoporosis on osteoarthritis.

Exposure-outcome	No. of SNP	IVW			Weighted median		Weighted mode		Simple mode		MR-Egger		MR-Raps	
		Beta (95%CI)	OR (95%CI)	P value	Beta (95%CI)	P value	Beta (95%CI)	P value	Beta (95%CI)	P value	Beta (95%CI)	P value	Beta (SE)	P value
OP-Total OA	16	-0.285 (-0.445, -0.126)	0.751 (0.640, 0.881)	4.41e-4	-0.260 (-0.480, -0.040)	0.020	-0.250 (-0.625, 0.125)	0.211	-0.184 (-0.564, 0.196)	0.357	-0.581 (-1.391, 0.228)	0.181	-0.289 (0.086)	7.99e-4
OP- KOA	16	-6.11 (-9.88, -2.34)	0.002 (5.07e-5, 9.56e-2)	1.47e-3	-4.60 (-8.46, -0.736)	0.019	-4.04 (-10.39, 2.30)	0.230	-5.50 (-12.61, 1.60)	0.149	-2.92 (-22.64, 16.79)	0.775	-5.69 (2.06)	5.75e-3
OP-HOA	16	-5.48 (-9.21, -1.74)	4.15e-3 (9.93e-5, 0.173)	3.99e-3	-5.54 (-10.13, -0.960)	0.017	-6.04 (-13.67, 1.58)	0.141	-5.86 (-13.80, 2.07)	0.168	-14.77 (-33.74, 4.18)	0.148	-5.66 (2.07)	6.30e-3

OP, osteoporosis; OA, osteoarthritis; Se, standard error; SNP, single nucleotide polymorphism; MR, Mendelian randomization; IVW, inverse variance weighting.

**Table 2:** MR-PRESSO analysis and MR-Egger intercept of osteoporosis causally linked to osteoarthritis.

Exposure	Outcome	MR-PRESSO Global test-p value	Main MR results P value	Egger-intercept	intercept-P value
Osteoporosis	Total OA	0.817	7.44e-4	6.07e-4	0.477
	KOA	0.394	0.015	-6.5e-3	0.751
	HOA	0.212	0.011	0.019	0.343

OA, osteoarthritis; MR, Mendelian randomization.

**Table 3:** Heterogeneity tests of osteoporosis causally linked to osteoarthritis.

Exposure	Outcome	IVW		MR-Egger	
		Cochran’s Q	Q- P value	Cochran’s Q	Q- P value
Osteoporosis	Total OA	10.408	0.793	9.876	0.771
	KOA	32.040	0.006	31.802	0.004
	HOA	19.915	0.175	18.637	0.179

OA, osteoarthritis; MR, Mendelian randomization; IVW, inverse variance weighting.

### The causality between OP and KOA

**Figure 2:**
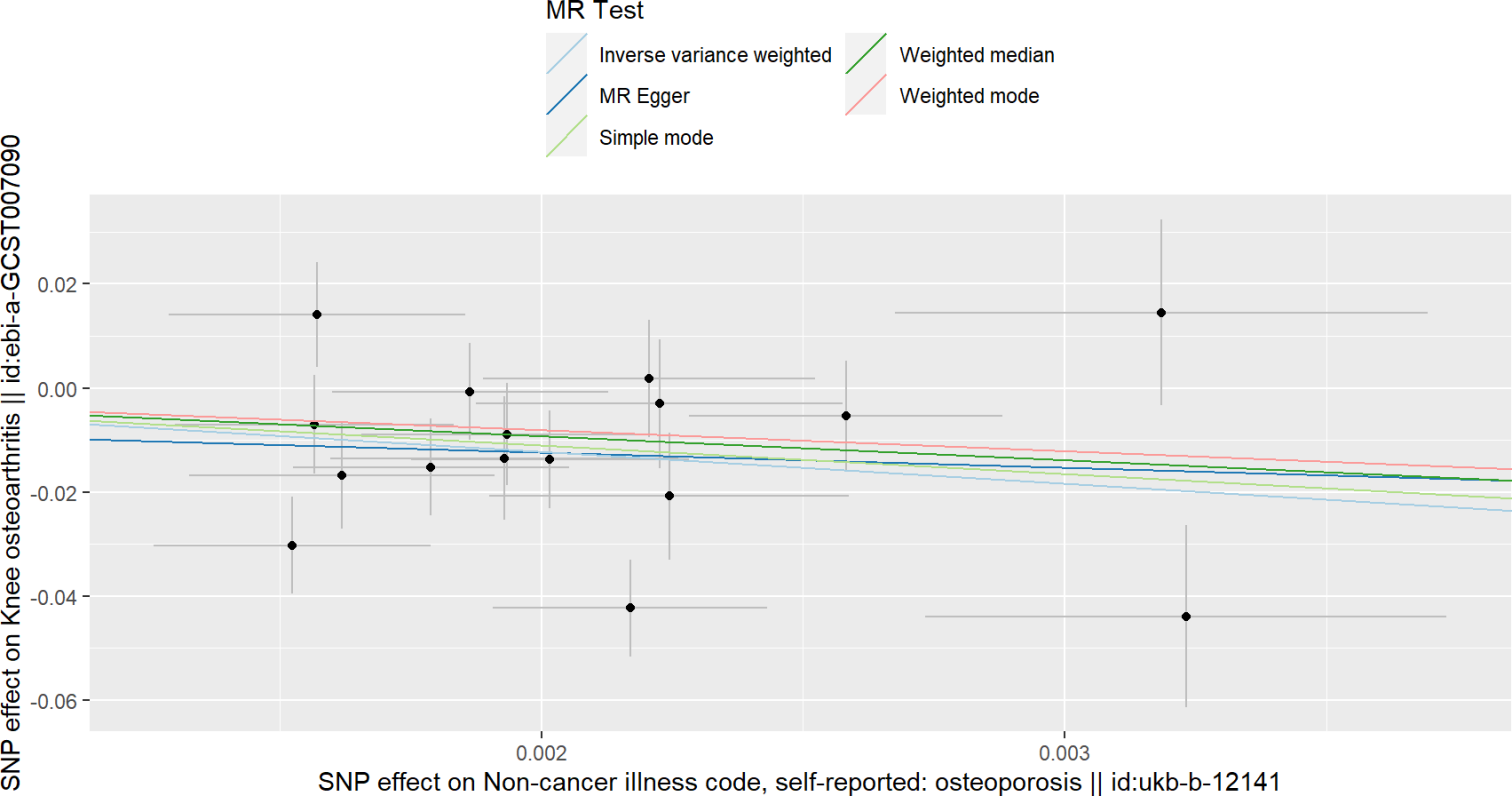
Scatterplots of the causal relationships between osteoporosis and knee osteoarthritis.

**Table 1** contains the MR estimates of different approaches employed to assess the causal effects of OP on KOA, indicating that there is a negative correlation between OP and KOA at the level of genetic prediction (IVW: Beta=-6.11, OR=0.002, *P* value< 0.016; Weighted median: Beta=-4.60, *P* value=0.018; MR-RAPS:beta=-5.69, *P* value=5.75e-3) (**Figure 2**). However, the results of Weighted mode, Simple mode and MR-Egger were not significant (**Table 1**). The results of MR-PRESSO analysis along with the results of the MR-Egger intercept analysis show that there is no horizontal pleiotropy in this analysis (the P values of the two tests were both greater than 0.05) (**Table 2**). However, heterogeneity analysis showed that there was heterogeneity in the IVs included in the analysis (the *P* values of Cochran’s Q tests of IVW and MR-Egger were both less than 0.05) (**Table 3**). Although there was heterogeneity in this analysis, the results of MR-RAPS analysis were consistent with the results of IVW. There was no SNP that affected the stability of the results (**Supplementary File 2**; **Figure S5**). Based on the results of IVW, horizontal pleiotropy test, retention method analysis and MR-RAPS, we considered that OP was a protective factor against KOA. For the forest map of this analysis, see **Supplementary File 2**; **Figure S2**.

### The causality between OP and HOA

**Figure 3:**
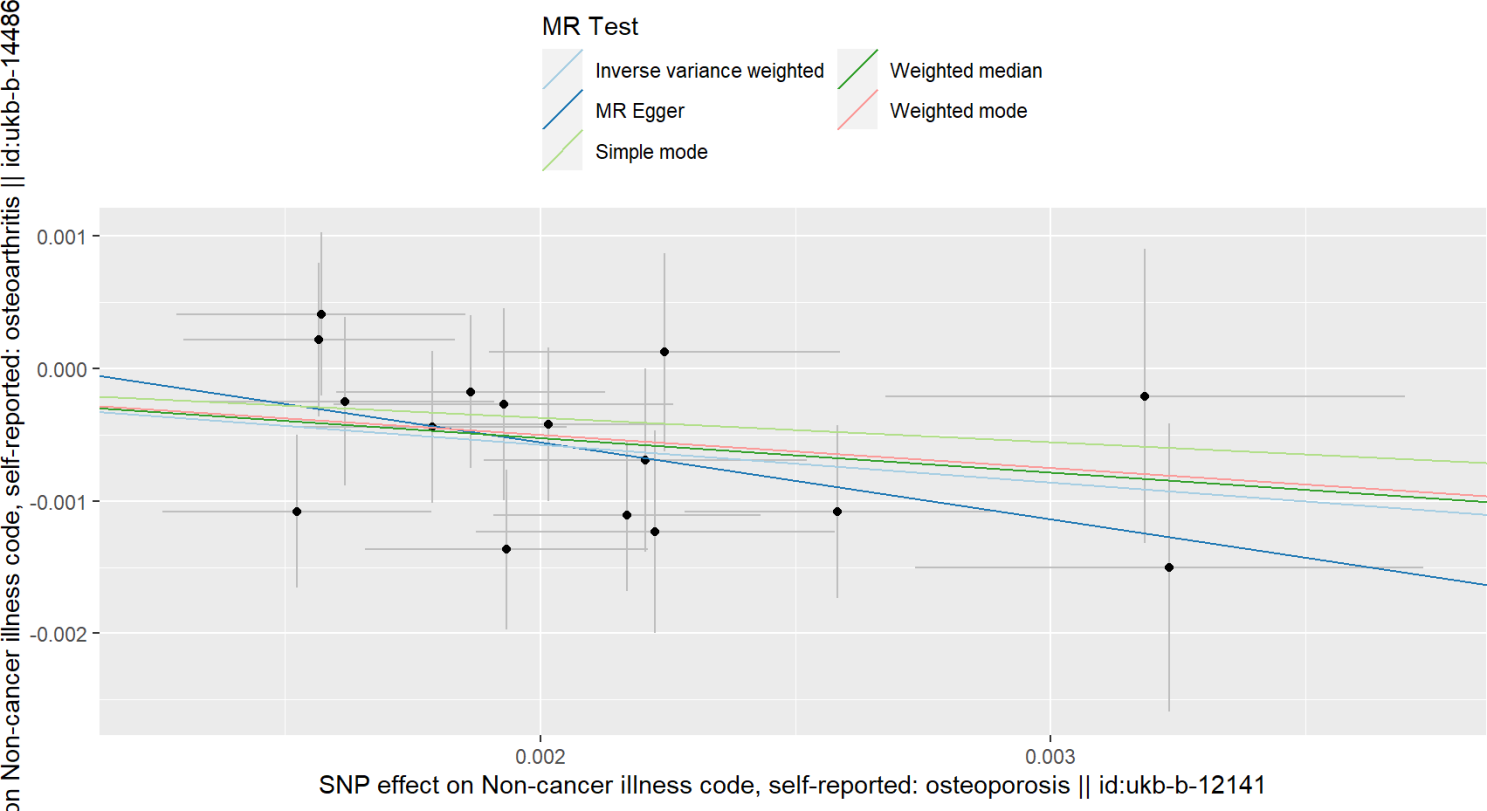
Scatterplots of the causal relationships between osteoporosis and hip osteoarthritis.

The IVW results in **Table 1** show that there is a causal relationship between OP and HOA, and OP has a protective effect on HOA (beta=-5.48, OR=4.15e-3, *P* value= 3.99e-3) (**Figure 3**). In addition, the results of weighted median and MR-RAPS are consistent with the results of IVW (**Table 1**). However, the results of Weighted mode, Simple mode and MR-Egger were not significant (**Table 1**). No positive results were found in the horizontal pleiotropy test or heterogeneity analysis (the *P* values of MR-PRESSO analysis, MR-Egger intercept analysis and Cochran’s Q were all greater than 0.05) (**Tables 2**, **3**). The forest map in this analysis is shown in **Supplementary File 2**; **Figure S3**. The leave-one-out test did not find problematic SNPs, as shown in **Supplementary File 2**; **Figure S6**. According to the above results, since no horizontal pleiotropy exists in the analysis, we regard IVW as the main criterion of causality, so we think that the genetically predicted OP is the protective factor of HOA.

## Discussion

Based on the genetic data from the open database, we conducted an MR analysis of whether OP is associated with the risk of OA. Based on our MR analysis, OP possessed negative correlations with the risk of total OA, KOA and HOA. The results we found are consistent with some previous clinical and genetic studies (8). For example, a cross-sectional study consistently found that osteoporosis was negatively correlated with osteoarthritis (OA), while bone mineral density (BMD) possessed positive correlations with knee OA and hip OA (6, 8, 23). In addition, genetic studies on the association of BMD with OA have also found that an increase in BMD increases the risk of OA (24). Our study is the first to find that OP is negatively related to the risk of OA from a genetic point of view.

One of the pathological features of OP is a decrease in BMD. At present, evidence of causality in high BMD of KOA has been observed (5, 25). The risk of KOA is positively correlated with the level of BMD. Our results show that OP can reduce the risk of KOA; that is, with a decrease in BMD, the risk of KOA also decreases. Previous studies have observed that high bone mass is associated with osteophyte volume (26), imaging OA (27, 28) and increased joint replacement rate (29). In addition, several studies have observed a stronger association between BMD and osteophytes than with JSN (30), which may be because the repeated stress on the joint surface increases with the increase in BMD, and these pathological changes make the articular cartilage vulnerable to mechanical erosion (31), resulting in cartilage damage. In addition, with the development of OA, the bone turnover of subchondral bone increases, and abnormal bone formation and hardening enhance the strength and stiffness of subchondral bone but reduce the ability of elastic deformation and shock absorption, resulting in unbalanced mechanical stress distribution or overload in articular cartilage, which in turn leads to articular cartilage wear due to joint movement (32, 33). In OP patients, the decrease in bone mineral density increases the elasticity and shock absorption capacity of bone tissue, which may reduce the progression of OA. The bone mineral density is proportional to the stiffness, and the increase of the stiffness of the subchondral plate will weaken its ability as an overlying cartilage shock absorber (34). In addition, according to genetic studies, OP and OA may have common genetic factors. For example, a matched case-control study found that the bone mineral density of the femoral neck of twins with hip osteophytes was higher than that of unaffected twins (23). The study also showed a general increase in bone mineral density in patients with osteoarthritis, indicating a common genetic factor of hip osteoarthritis and high bone mass (23).

Arokosky et al. reported that radiological hip OA patients had significantly higher femoral neck width (volume) and BMD than healthy controls mapped with age and sex (35). In a matched case-control study, twins with hip osteophytes were found to have higher BMD of the femoral neck than unaffected twins in twins with inconsistent OA conditions (23). In addition, the study showed a general increase in BMD in those patients with OA, suggesting that HOA and high bone mass share a common genetic factor. Our study found that OP can reduce the incidence of HOA, which proves the causal relationship between HOA and OP from a genetic point of view and reduces the heterogeneity and environmental bias of observational studies. But the researchers found that increased BMD did not increase the risk of OA in all areas, for example, there was no causal relationship between BMD and hand OA (24),which is due to the increased risk of weight-bearing OA due to joint load caused by weight, while non-weight-bearing joints such as hands are not affected.

Although previous studies have discussed the causal relationship between BMD and OA, our study is the first to use Mendelian randomized analysis to explore the causal relationship between OP and OA. Although the decline of BMD is a sign of OP, the performance of OP is not only the decline of BMD, so our study is the first time to use OP as an exposure variable to conduct a Mendelian randomized analysis. From the genetic point of view, OP has been proven to be able to reduce the incidence of KOA. In the current research, SNPs with genome-wide association and independent inheritance but having no LD were chosen as IVs to assess the causal relationship of OP with OA, which makes our results more reliable. In addition, the results of horizontal multiplicity analysis show that the use of these genetic tools will not lead to horizontal diversity. Although heterogeneity was found in the analysis of OP and KOA, the conclusion obtained by MR-RAPS analysis is consistent with the results of IVW, and the test results show that our results are robust.

Our results also have some limitations, such as our inability to assess the amount of overlapping data. However, the deviation of sample overlap is reducible by way of some tools (for instance, the F statistic is unusually larger than 10). In addition, the aggregated GWAS data are from populations of European ancestry, so our conclusions may not be applicable to other ethnic groups. In addition, some methods we used did not obtain the same conclusions as IVW, but the SNPS included in this study met the hypothesis of being effective instrumental variables, and the results of MR-RAPS were consistent with the results of IVW. The analysis of the other methods did not find that any SNPS would affect the stability of the results, so our conclusions were reliable.

## Conclusions

We explored the causal relationship between OP and OA through a two-sample MR study and found that OP can reduce the incidence of OA (including KOA and HOA). These findings explain the causal relationship between OP and OA from the perspective of genetics and provide new insights into the relationship between OP and OA in the future. Of course, our conclusions need to be proven by further clinical and basic experiments.

## Data availability statement

Data used in the present study are all publicly available. Authors will provide the data upon reasonable request.

## Author contributions

LL: writing-original draft. LL: WH and PL: conceptualization: project administration: and writing-review and editing. PL: JW and MY: data curation and methodology. PX and LL: formal analysis: validation: visualization and software. All authors contributed to the article and approved the submitted version.

## Funding

This work was supported by the National Natural Science Foundation of China (82072432).

## Conflict of interest

The authors declare that the research was conducted in the absence of any commercial or financial relationships that could be construed as a potential conflict of interest.

## Publisher’s note

All claims expressed in this article are solely those of the authors and do not necessarily represent those of their affiliated organizations, or those of the publisher, the editors and the reviewers. Any product that may be evaluated in this article, or claim that may be made by its manufacturer, is not guaranteed or endorsed by the publisher.
