# Supplementary material for: Causal relationship between osteoporosis and osteoarthritis: A two-sample Mendelian randomized study
Source: Front Endocrinol (Lausanne). 2022 Oct 21;13:1011246. doi: 10.3389/fendo.2022.1011246 (PMC9633945; doi:10.3389/fendo.2022.1011246)
Supplement: Supplementary file 2 [file DataSheet_1.docx]

**Figure S1: Forest plot of variant specific inverse variance estimates for the causal association between osteoporosis and total osteoarthritis**

**
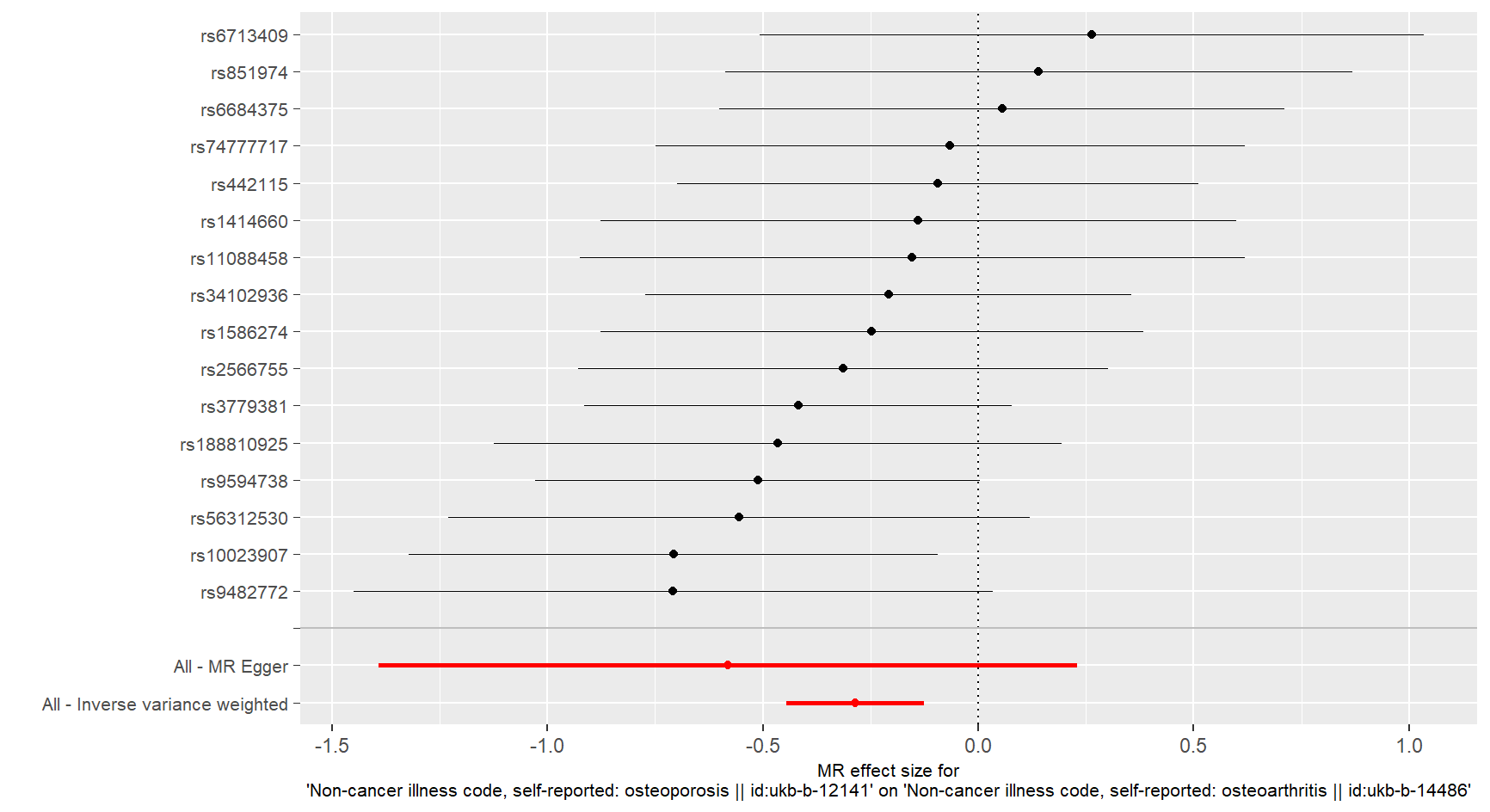
**

**Figure S2: Forest plot of variant specific inverse variance estimates for the causal association between osteoporosis and knee osteoarthritis**

**
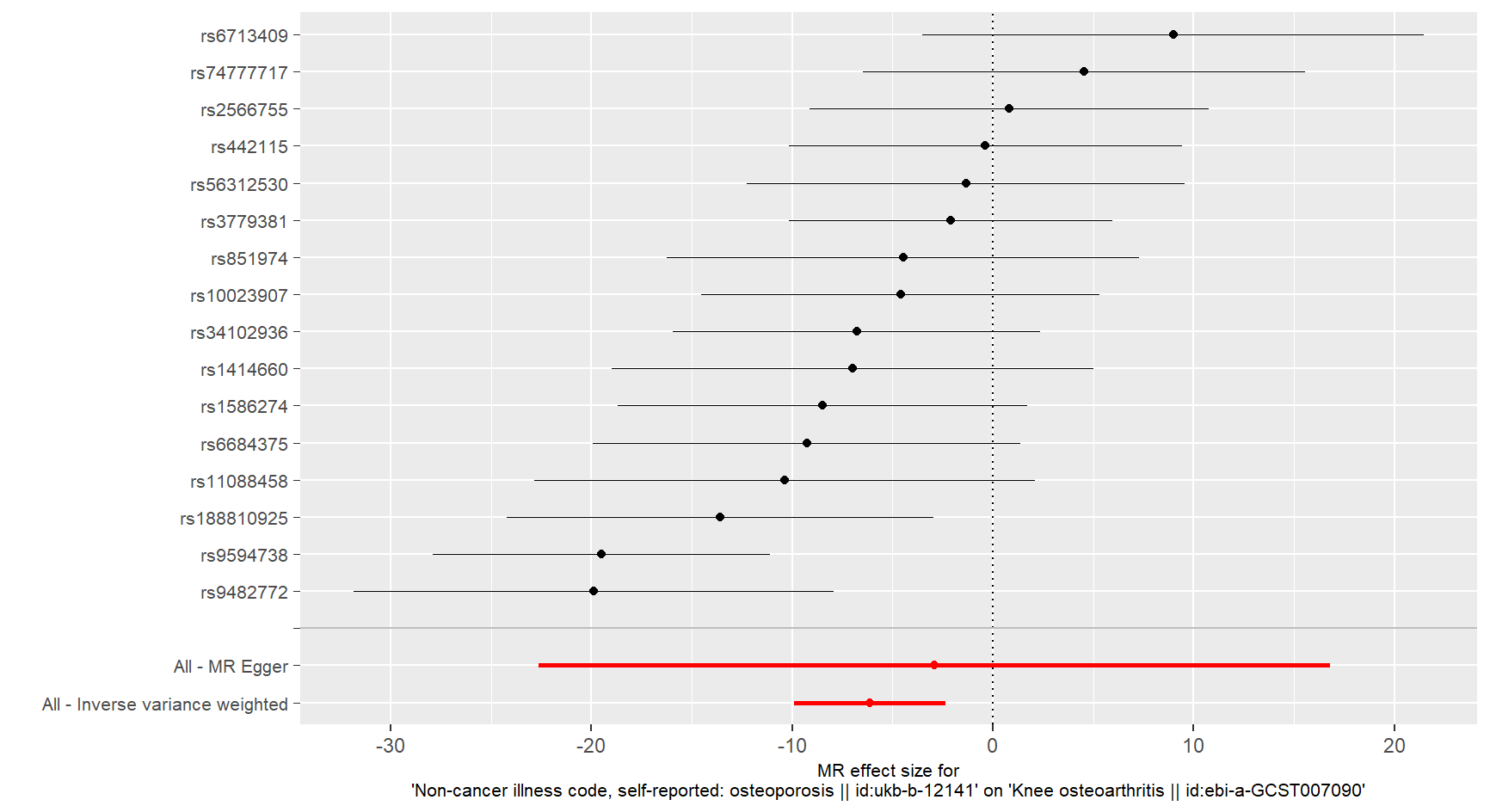
**

**Figure S3: Forest plot of variant specific inverse variance estimates for the causal association between osteoporosis and hip osteoarthritis**

**
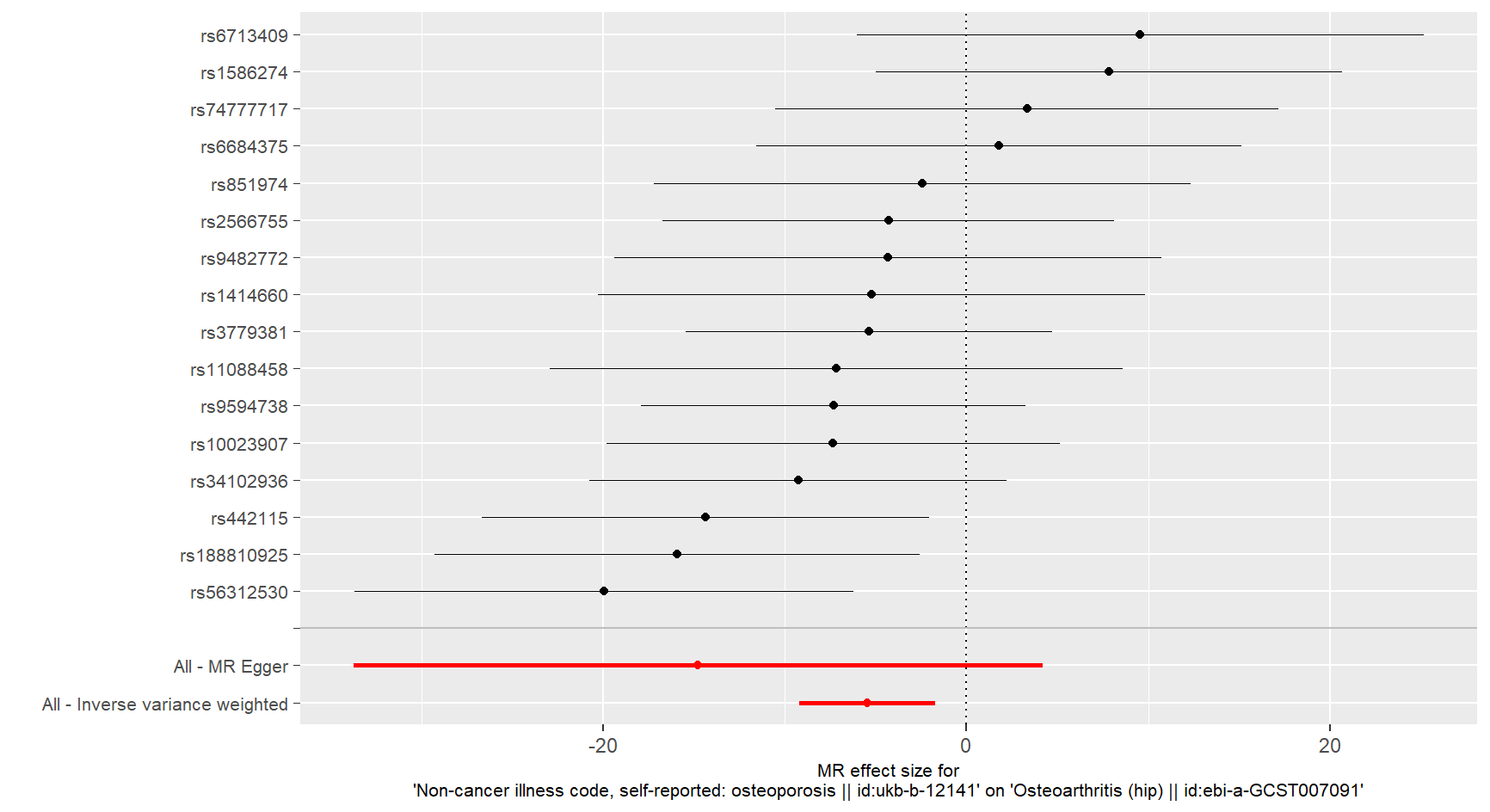
**

**Figure S4: Leave-one-out test plot of the causal association between osteoporosis and total osteoarthritis**

**
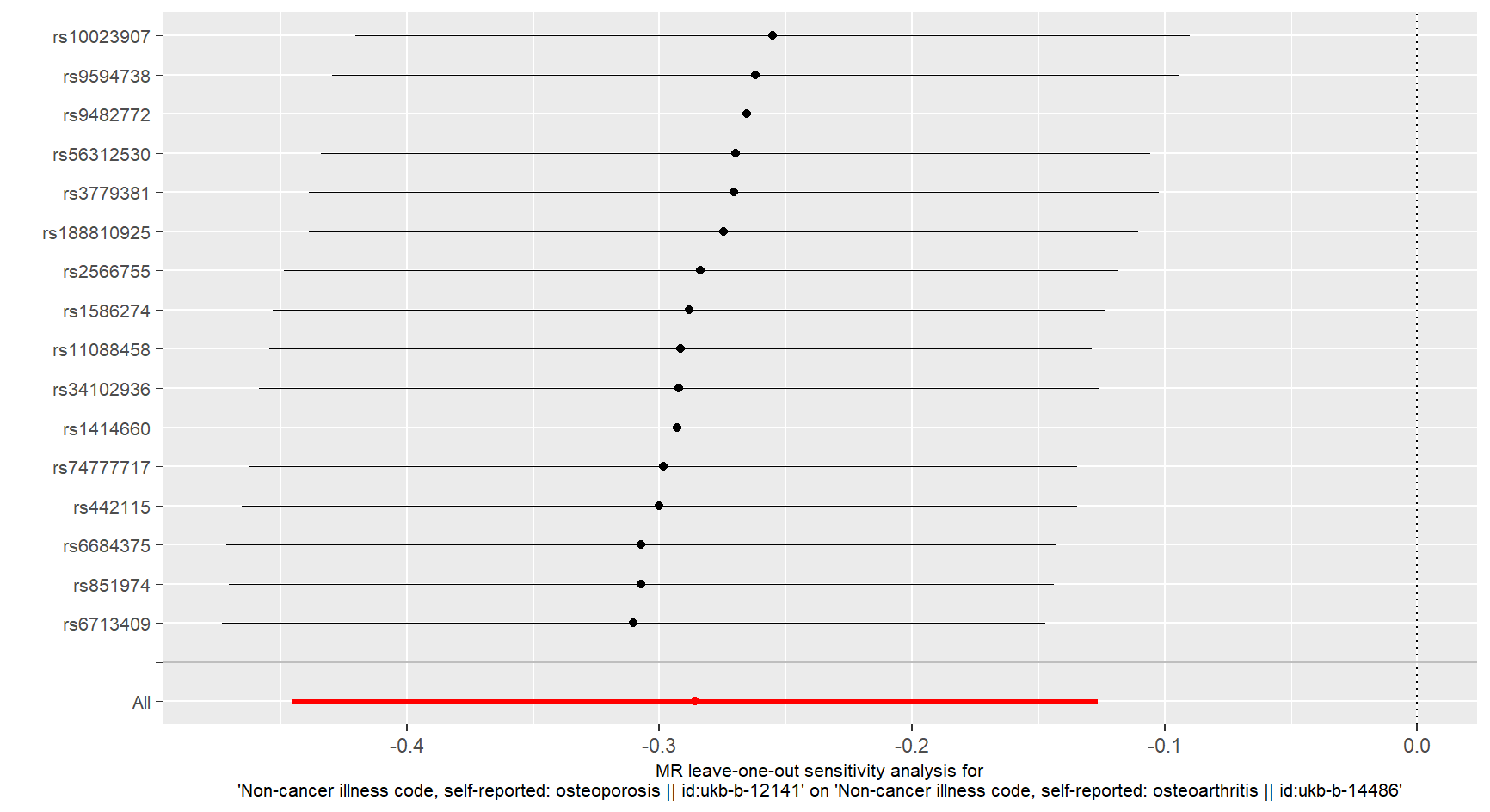
**

**Figure S5: Leave-one-out test plot of the causal association between osteoporosis and knee osteoarthritis**

**
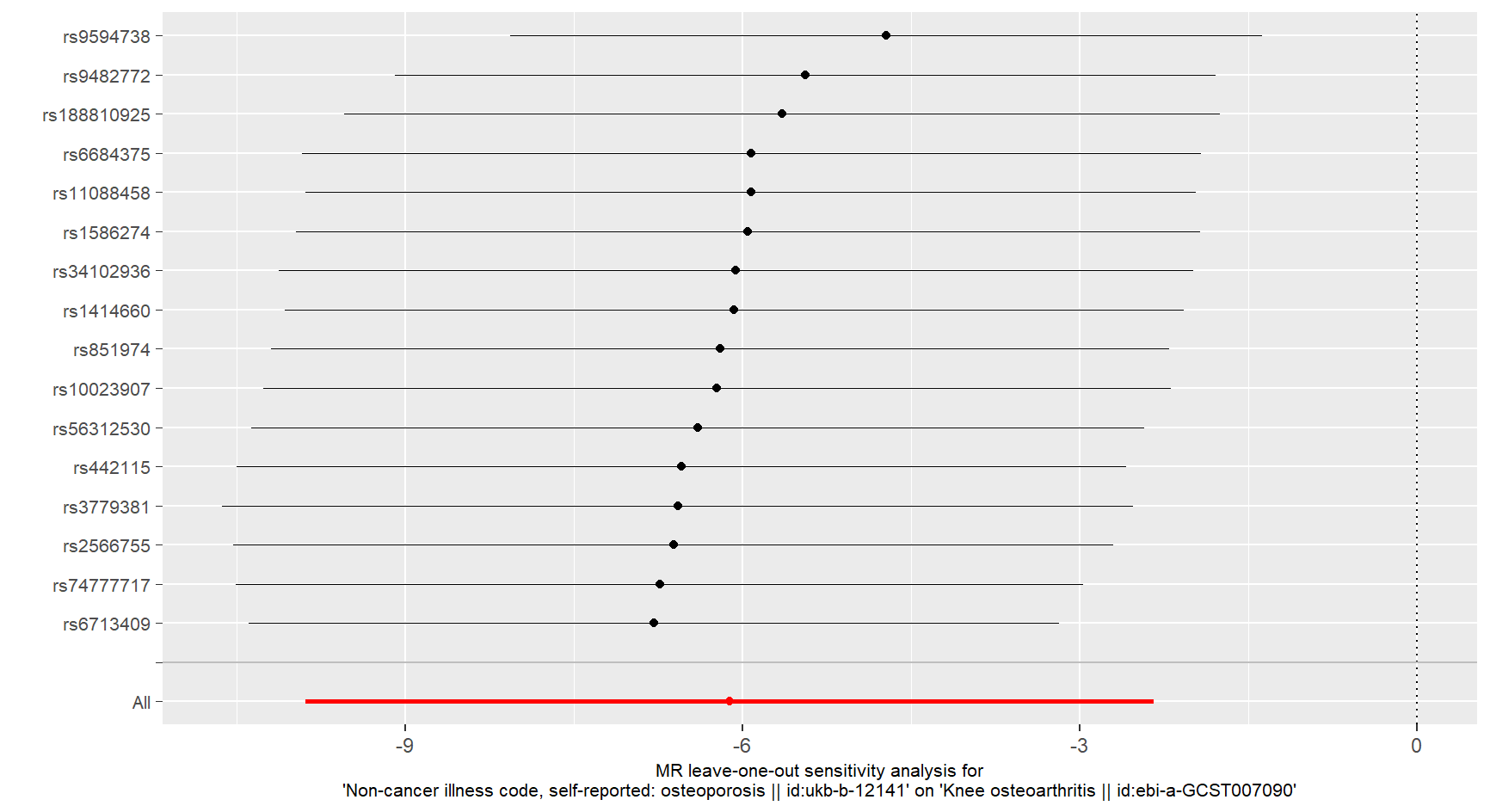
**

**Figure S6: Leave-one-out test plot of the causal association between osteoporosis and hip osteoarthritis**

**
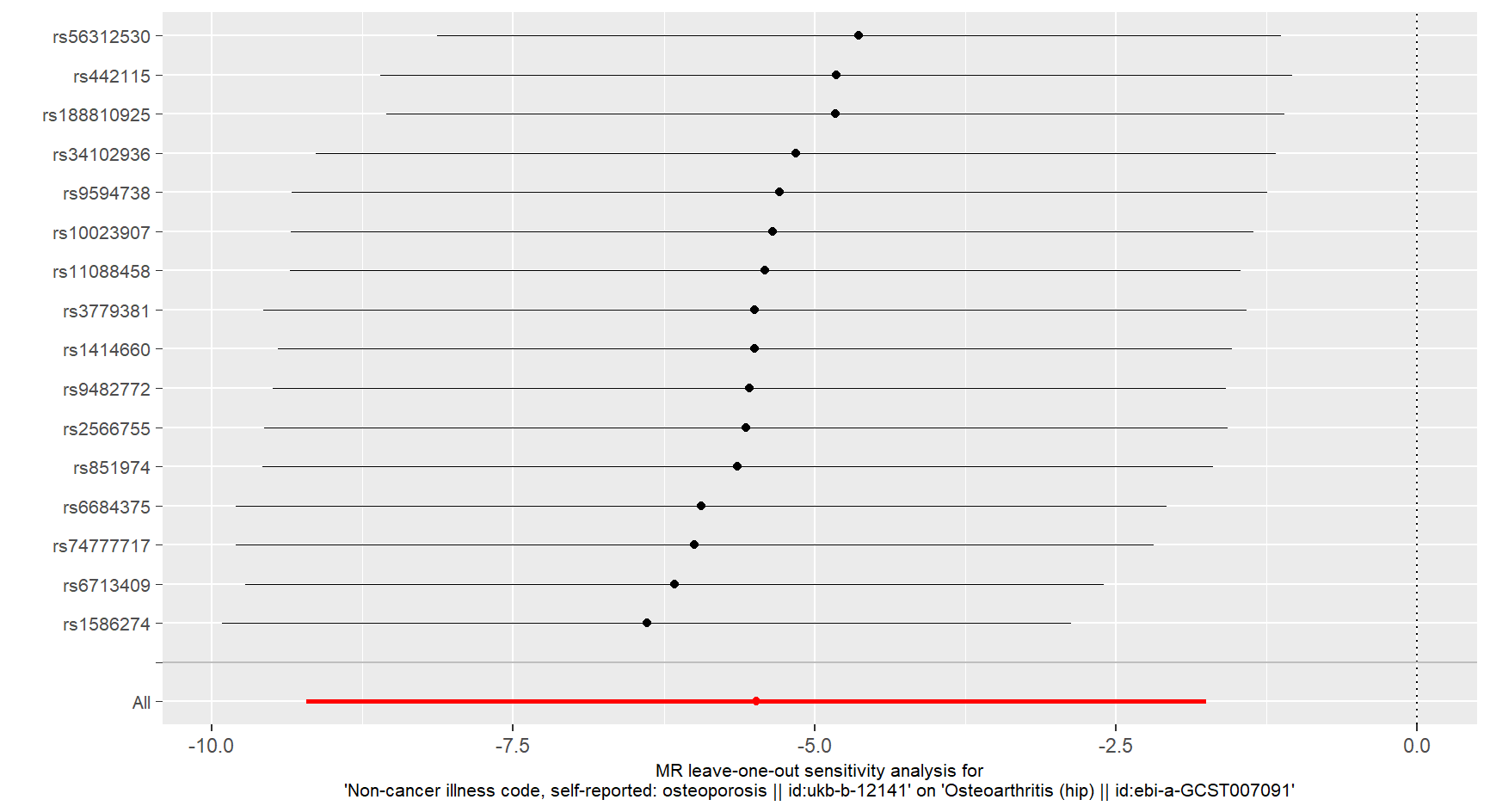
**
